# Human hunters are no substitute for vanishing apex predators

**DOI:** 10.1111/1365-2656.70306

**Published:** 2026-06-26

**Authors:** Ying Geng, Christos Mammides, Ru‐Chuan He, Lin Wang, Kang Luo, Zhong‐Yuan Zhang, Bo Wang, Rui‐Chang Quan

**Affiliations:** ^1^ Southeast Asia Biodiversity Research Institute, Chinese Academy of Sciences & Center for Integrative Conservation, Xishuangbanna Tropical Botanical Garden Chinese Academy of Sciences Mengla China; ^2^ University of Chinese Academy of Sciences Beijing China; ^3^ Scientific Research Institute of Xishuangbanna National Nature Reserve Jinghong Yunnan China; ^4^ CAS Key Laboratory of Tropical Forest Ecology, Xishuangbanna Tropical Botanical Garden Chinese Academy of Sciences Mengla China

**Keywords:** apex predator ecological function, biodiversity conservation, bipartite network stability, camera trap, hunter super‐predator, richness, species composition, wildlife management

## Abstract

1. The global decline of apex predator populations risks the loss of their crucial ecological functions. This raises a pressing yet contentious question: can human hunters, often termed ‘super‐predators’, functionally substitute for the complex regulation once provided by their natural counterparts?
2. To investigate this question, we analysed 30,778 camera‐trap records from 400 sites in southwestern China (2017–2021). Using propensity score matching (PSM) to control for environmental heterogeneity, we compared wildlife communities across hunter‐dominated, apex predator‐dominated, and predator‐absent sites.
3. Our results showed that hunters fail to replicate the collective and individual ecological functions of natural apex predators (dhole, Asian golden cat, and clouded leopard). Apex predator sites supported the highest species richness and abundance, with 33.0% and 32.5% more species and 49.8% and 44.8% greater abundance than hunter‐dominated and predator‐absent sites, respectively. The prey species–site network was the most robust at apex predator sites and the weakest at hunter‐dominated sites, indicating that hunting increases prey vulnerability to cascading extirpations following habitat loss.
4. Compared with hunter‐dominated sites, sites dominated by single‐apex predators had distinct species compositions and dominant prey. Prey exhibited prolonged avoidance (up to 2.6 times longer) of hunters compared with any apex predator, coinciding with the weakest network robustness at hunter‐dominated sites.
5. Collectively, our findings provide compelling evidence that human hunters cannot replace apex predators in sustaining biodiversity and promoting stable spatial patterns. Our work therefore strongly supports the conservation of natural apex predators and offers crucial insights for regulating human hunting in ecosystem management.

## Graphical abstract

Our study reveals that human hunters fail to replicate the collective and individual ecological functions of natural apex predators in sustaining biodiversity and promoting stable spatial patterns. These insights are vital for rethinking predator conservation and wildlife management in human‐dominated landscapes.

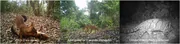

## 1 INTRODUCTION

Apex predators, although integral to ecosystem regulation, have suffered drastic declines in recent centuries due to overhunting, habitat loss, and human–wildlife conflict (Ripple et al., 2014). These declines have compromised their role in top‐down regulation of lower trophic levels, leading to cascading ecological consequences, including herbivore overabundance, mesopredator release, and overall community destabilization (Berger et al., 2001; She et al., 2023). Despite efforts to reintroduce apex predators, their recovery has proven challenging due to barriers such as human–wildlife conflict, policy constraints, and public opinion (Liu et al., 2024; Morell, 2022). As apex predators vanish and their functions diminish even before extinction (Säterberg et al., 2013), the ecological role of human hunters is increasingly being reconsidered (e.g., Cassinello, 2021; She et al., 2023).

In the Anthropocene, human hunting has become globally pervasive across trophic levels, positioning hunters as super‐predators (Darimont et al., 2015). Historically, hunting, especially the extraction of bushmeat, has been regarded as a potentially unsustainable practice (Brodie et al., 2015; Harrison et al., 2016). The introduction of firearms, in particular, greatly increased hunting efficiency and facilitated the killing of large animals (Kümpel et al., 2008; Levi et al., 2011), leading to widespread population declines, local extirpations, and even species extinctions (Benítez‐López et al., 2019). However, ecologists now acknowledge that hunters can potentially play important and varied roles in communities. For example, given their ability to regulate animal populations, hunters may contribute to food web and community recovery (Cassinello, 2021; She et al., 2023) and support complex trophic interactions (Berger et al., 2001). This shift raises a central question: can hunters serve as effective substitutes for natural apex predators?

Research addressing this question has produced contrasting results, primarily centred on two mechanisms of top‐down regulation exerted by apex predators: density‐mediated effects and trait‐mediated effects (Creel & Christianson, 2008). The former involves direct predation that regulates prey population size, while the latter refers to the ‘landscape of fear’, whereby predators reorganize prey behaviour and distribution through non‐lethal means. Proponents argue that targeted hunting can regulate populations of specific species, such as white‐tailed deer (*Odocoileus virginianus*) in North America (McShea, 2012). Moreover, similar to natural apex predators, prey species also develop ‘fear’ of hunters (Clinchy et al., 2016; Liu et al., 2023). By leveraging this perceived risk, wildlife managers can manipulate ungulates' spatial distributions to deter them from specific areas, thereby mitigating human–wildlife conflicts (Cromsigt et al., 2013).

However, some ecologists argue that human hunting strategies are often more unpredictable and intensive than those of natural apex predators. Human hunting is largely decoupled from biological constraints such as environmental conditions, nutritional needs, or prey size (Lennox et al., 2022; Sala et al., 2018). Consequently, hunters exhibit significant spatial flexibility, alternating between selective trophy hunting (Darimont et al., 2015) and opportunistic exploitation of chance encounters (Dalerum & Swanepoel, 2017). In contrast, natural predators are governed by environmental cues, such as diel cycles (Palmer et al., 2022), and must optimize their activity based on energetic costs. Moreover, human hunting often occurs in high‐intensity pulses within narrow temporal windows, whereas apex predators typically maintain a persistent, year‐round presence (Wilmers et al., 2003), exerting more consistent and spatially distributed regulatory pressure.

Given these distinct predation regimes, the impacts of apex predators and hunters may be asymmetric across taxa. Populations of common species (e.g., highly reproductive ungulates) may recover more rapidly following disturbance (Steinmetz et al., 2010). In contrast, rarer species, particularly small‐bodied carnivores, often exhibit heightened sensitivity to human presence (Clinchy et al., 2016) and may prioritise spatiotemporal avoidance as a primary response (Palmer et al., 2022). In multi‐predator systems, diverse hunting modes—exemplified by the solitary ambush strategy of cougars (*Puma concolor*) versus the cooperative pursuit of grey wolves (*Canis lupus*)—provide functional complementarity (Kays & Wilson, 2009). This diversity fosters complex prey‐regulation dynamics (Atwood et al., 2009), thereby enhancing overall ecosystem stability. However, it remains unclear whether hunters can functionally substitute for apex predators, particularly within complex multi‐predator systems, and how prey behavioural responses diverge across hunter and apex predator predation regimes.

Therefore, evaluating the functional equivalence of hunters and apex predators requires a multi‐scale comparison of their influences on individual behavioural patterns and community‐level diversity. We utilized an extensive camera‐trap dataset from Xishuangbanna Dai Autonomous Prefecture (hereafter Xishuangbanna), Yunnan, China, a biodiversity hotspot where apex predators such as large felids and dholes (*Cuon alpinus*) have been largely extirpated or have become rare (Chen et al., 2019). Despite strict regulations banning private firearm possession and the hunting of protected species (Li, 2007), illegal hunting (poaching) persists among indigenous communities in Xishuangbanna, leading to significant biodiversity loss (Huang et al., 2020). However, the shift in agriculture, particularly the expansion of rubber cultivation, which has considerably improved the economic status of the locals (Xu et al., 2014), has changed the primary motive for hunting from subsistence to recreation (Chang et al., 2017). While hunters continue to mostly target ungulates (e.g., *Sus scrofa*, *Muntiacus vaginalis*), smaller carnivores (e.g., *Paradoxurus hermaphroditus*, *Paguma larvata*) (Chen et al., 2019), and birds (Commerçon et al., 2021), the transition towards leisure hunting has made the practice more opportunistic. With over a third of hunters (36.7%) having previously expressed intent in continuing this activity (Chang et al., 2017), its effect across different taxonomic groups is becoming increasingly difficult to predict. This decline of apex predators, coupled with persistent hunting pressure, provides a unique opportunity to assess whether hunters can functionally compensate for the loss of natural apex predators. To examine this, we compared the ecological roles of hunters and natural apex predators across two scales: (1) Hunters vs. Apex Predator Guild: how hunters and apex predators shape community structure through effects on species richness, abundance, and prey spatial distribution; and (2) Hunters vs. Individual Apex Predator: how hunters and individual apex predators (dhole, Asian golden cat, and clouded leopard) differ functionally in regulating species composition, maintaining prey anti‐predator behaviours, and ensuring robust spatial regulation.

## 2 MATERIALS AND METHODS

### 2.1 Study area

**FIGURE 1:**
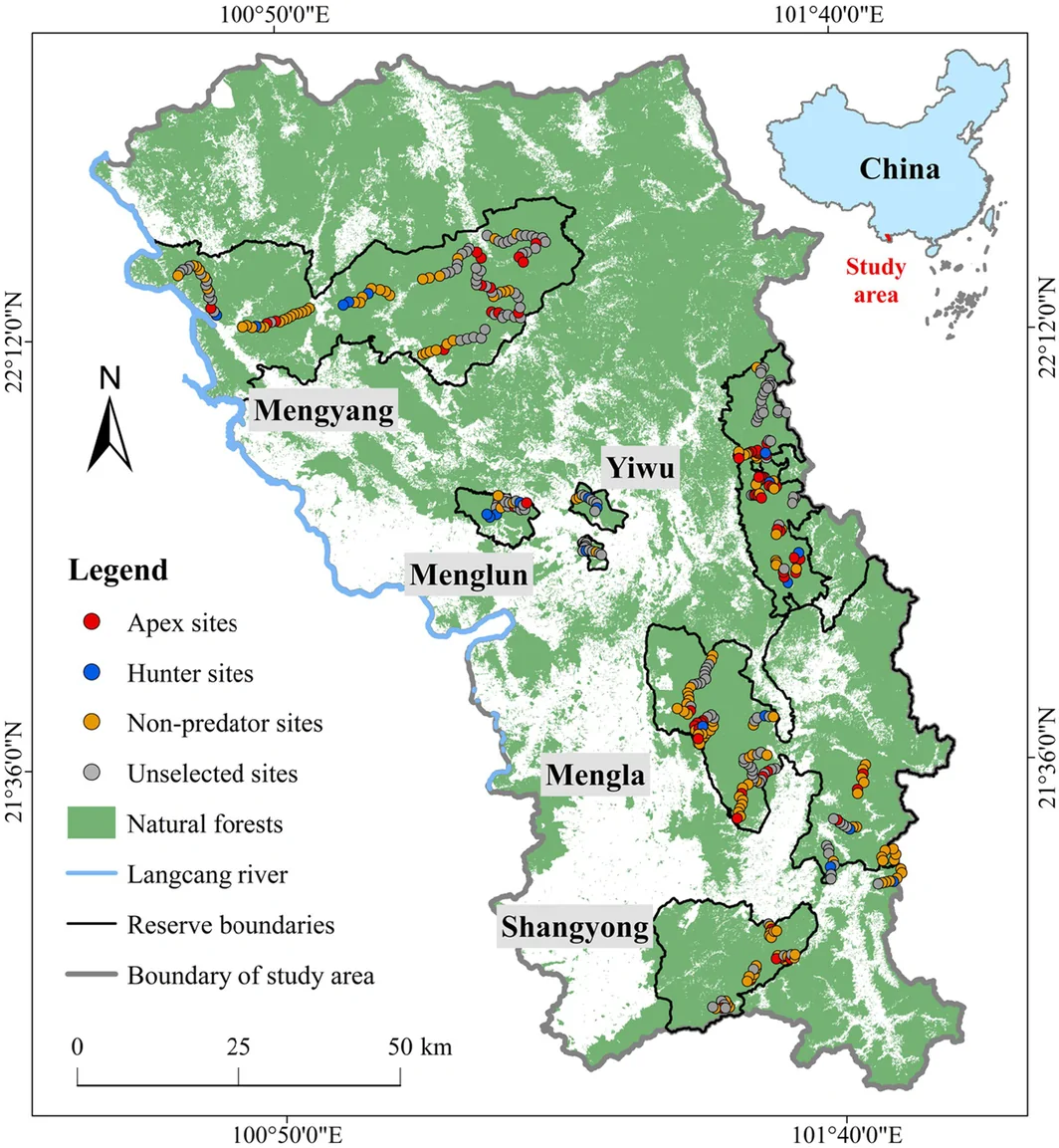
Camera‐trap sites in the study area (eastern Xishuangbanna, Yunnan Province, China). The map displays all 400 initial camera‐trap stations. The 236 sites selected for the final analysis are colour‐coded (red, blue, and orange) based on predator distribution and were matched using propensity score matching to ensure environmental comparability (see ‘Site Selection’ for details). Excluded sites are indicated by grey circles.

Our study was conducted in the eastern part of Xishuangbanna, Yunnan Province, southwestern China (Figure 1). Divided by the Lancang River, this region is a vital habitat for large carnivores (Liu et al., 2025), encompassing both the Xishuangbanna National Nature Reserve (with four sub‐reserves: Mengyang, Menglun, Mengla, and Shangyong) and the Yiwu Prefectural Nature Reserve.

### 2.2 Camera‐trap survey

From 2017 to 2021, we used camera traps (Loreda L710, Shenzhen, China) to monitor mammal communities in the Xishuangbanna nature reserves. A total of 400 sites were established along transects, including locations in both core and edge areas. Each camera was placed at least 500 m apart from other cameras (e.g., He et al., 2024; Lwin et al., 2023). Cameras were mounted on tree trunks at a standardized height of 0.5 m. Each trigger produced three consecutive images. Batteries and memory cards were replaced every 6 months (He et al., 2024). Although each camera was intended to operate for 1 year, recovered cameras functioned for an average of 284 days (SD: 78; range: 77–379) due to damage or loss (Table S1). All the camera‐trap surveys in this study were approved by the State Forestry Administration of China (No. 439). As a non‐invasive study relying solely on camera‐trapping data, no animal capturing or handling was conducted, and no ethical approval was required.

### 2.3 Study animals

We focused on medium‐ and large‐sized wildlife, typically targeted by hunters (Corlett, 2007), including mammals ≥1 kg and ground‐dwelling birds ≥0.2 kg. Camera traps also recorded humans and livestock; individuals carrying firearms were classified as hunters (Figure S1), whereas those without firearms were classified as local villagers (Ferreguetti et al., 2018). Following Carbone and Gittleman (2002), we defined natural apex predators as species with large body size, a carnivorous diet, low density, and population dynamics primarily driven by prey availability. Based on this definition, the apex predators considered here were the dhole (*Cuon alpinus*), Asian golden cat (*Catopuma temminckii*), and clouded leopard (*Neofelis nebulosa*) (Figure S1).

We quantified the relative abundance of all animals, hunters, villagers, and livestock using the relative abundance index (RAI), defined as the number of independent detections per 100 camera‐trap days (He et al., 2024; Lwin et al., 2023). Two detections were classified as independent events (IEs) if they consisted of consecutive photographs of the same species taken more than 30 min apart (O'Brien et al., 2003). Species rarity was assessed using an abundance–occupancy space approach (Li et al., 2020). For each species, we calculated its average relative abundance as the mean RAI across all camera sites, including sites with zero detections. Similarly, species‐specific occurrence frequency was calculated as the proportion of sites at which the species was detected at least once (Li et al., 2020). Following Li et al. (2020), species with both high abundance and high occurrence were considered prevalent, whereas those with low RAI and limited occurrence were considered rare. For analysis, species were classified into three groups: prevalent (occurring in >60% of sites with mean RAI >1.0), rare (occurring in <10% of sites with mean RAI <0.05), and common (all others) (Supporting Information‐I; Table S2).

### 2.4 Data analysis

#### 2.4.1 Site selection

To assess the effects of predation pressure on species richness, abundance, composition, and distribution, we divided the 400 camera sites into three categories based on the distribution of natural apex predators and hunters: (1) Apex Sites (*n*_sites_ = 81), where apex predators were detected but hunters were absent; (2) Hunter Sites (*n*_sites_ = 21), where hunters were detected but apex predators were absent; and (3) Non‐predator Sites (*n*_sites_ = 288), the control group, comprising sites where neither hunters nor apex predators were detected. Additionally, we identified sites where both hunters and apex predators were present (*n*_sites_ = 10). However, given the small number of such sites and the difficulty of disentangling the effects of each predator group on prey species due to the cumulative predation pressure, these sites were excluded from subsequent analyses.

To isolate the effects of predation risk from confounding environmental factors, we selected sites with similar environmental characteristics based on three groups of variables (Li et al., 2025): (1) landscape structure—tree density, normalized difference vegetation index (NDVI), slope, aspect, and topographic position index; (2) anthropogenic disturbance—distance to roads (town/village‐ and county‐level), distance to plantations (rubber and tea), and RAIs of villagers and livestock; and (3) key resources—distance to water surfaces. All spatial variables, except tree density (available at 1 × 1 km resolution), were rasterized at 30 × 30 m and processed in ArcMap 10.6. Distances were calculated using the ‘Near’ function. Detailed information on the spatial variables is provided in He et al. (2024).

To minimize environmental bias across the three site categories (Hunter, Apex, and Non‐predator), we first employed pairwise Wilcoxon rank‐sum tests (‘stats’ package, *pairwise.wilcox.test* function; R Core Team, 2025) to assess potential differences. Initial comparisons revealed no significant environmental differences between Hunter and Non‐predator sites (*p* > 0.05). However, significant differences were detected in the following comparisons: Hunter vs. Apex sites—tree density, distance to roads, and distance to plantations; and Apex vs. Non‐predator sites—tree density, distance to water surfaces, distance to plantations, and villager RAI. To resolve these imbalances, we implemented an iterative balancing procedure using PSM (‘MatchIt’ package, *matchit* function; Ho et al., 2011) with a nearest‐neighbour, no‐replacement algorithm. The matching process did not fully eliminate disparities, as significant differences remained regarding water proximity and villager RAI (*p* < 0.05). Therefore, we refined the matching by sequentially removing non‐predator sites with extreme values until balance was achieved (Supporting Information‐II). Post‐selection density plots confirmed a high degree of overlap in the distributions of environmental factors (Figure S2), and the final pairwise Wilcoxon tests confirmed no significant differences (*p* > 0.05) among the three site categories: hunter (*n*_sites_ = 21), matched apex (*n*_sites_ = 65), and non‐predator sites (*n*_sites_ = 150) (Table S3). Moreover, to ensure the independence of our observations, we used Moran's Index (‘spdep’ package, *moran. test* function; Bivand & Wong, 2018) to quantify the spatial autocorrelation of hunter and apex predator presence across the sampling sites. The results revealed no significant spatial autocorrelation for either apex predators or hunters (Table S4), thereby confirming that detections among sampling sites were independent.

Within the 65 apex sites, 55 hosted only one of the three apex predators (dhole, Asian golden cat, or clouded leopard). Co‐occurrence of apex predators was rare: nine sites hosted two species, and only one hosted all three. For comparisons between hunters and all apex predators, the three species were grouped into a single functional unit across all 65 sites. For analyses of individual roles, only the 55 single‐predator sites were used, enabling species‐specific effects to be isolated.

#### 2.4.2 Functional comparison between human hunters and the apex predator guild

To compare species richness and RAIs among hunter, apex, and non‐predator sites, we used Kruskal–Wallis rank‐sum tests (‘stats’ package, *kruskal.test* function; R Core Team, 2025), followed by post hoc multiple comparisons using Dunn's tests based on rank sums (‘dunn.test’ package, *dunn.test* function; Dinno, 2024). To examine the spatial distribution patterns of prey species in areas with hunters, apex predators, or no predators, we performed a bipartite network analysis (‘bipartite’ package, *networklevel* function; Dormann et al., 2009). We constructed three species–site matrices containing only prey species, corresponding to sites occupied by hunters, apex predators, or no predators, with each cell filled by the RAI of a prey species. To assess differences in network structure, we performed 30 bootstrap iterations for each network category: hunter sites (n_IEs_ = 1356), apex predator sites (n_IEs_ = 5960), and non‐predator sites (n_IEs_ = 10,012) (Wang et al., 2022).

We quantified network structure using the ‘robustness (high level)’ index, which measures the proportion of species that persist after the sequential removal of a given fraction of sites (Dormann et al., 2009). Higher robustness indicates that most species survive even after the loss of many sites, reflecting a balanced spatial distribution, high site redundancy, and stronger resistance to site loss. Lower robustness implies a fragile system in which the elimination of even a small number of sites can cause widespread extinction due to species' dependence on a few ‘refuge sites’, indicating concentrated distributions, limited alternative habitats, and lower community resistance (Dormann et al., 2009). We compared robustness metrics using a permutation analysis of variance (‘lmPerm’ package, *aovp* function; Wheeler & Torchiano, 2025), followed by pairwise permutation tests (‘rcompanion’ package, *pairwisePermutationTest* function; Mangiafico, 2025).

#### 2.4.3 Functional comparison between human hunters and individual apex predators

To assess compositional differences between single‐apex predator and hunter sites, we conducted a non‐parametric analysis of similarity (ANOSIM) using Bray–Curtis distances (‘vegan’ package, *anosim* function; Oksanen et al., 2025). Results were visualized using non‐metric multidimensional scaling (NMDS; ‘vegan’ package, *metaMDS* function; Oksanen et al., 2025). To identify the species contributing most to these dissimilarities, we performed a similarity percentage (SIMPER) analysis with 999 permutations for all pairwise site comparisons (‘vegan’ package, *simper* function; Oksanen et al., 2025).

We measured temporal avoidance to quantify differences in anti‐predator behaviour between hunters and individual apex predators. Temporal avoidance was defined as the time interval between a primary species' visit (hunter or apex predator) and the subsequent detection of a prey species at the same camera‐trap site (Niedballa et al., 2019). After identifying independent events for hunters and each of the three apex predator species, we calculated the elapsed time until the next prey detection at those sites. We analysed these time intervals using a linear mixed‐effects model (‘lme4’ package, *lmer* function; Bates et al., 2015). Log‐transformed avoidance time (to meet normality assumptions) was modelled with predator type (hunter and the three apex predators) as a fixed effect and prey species as a random effect to account for species‐specific variation. To further examine the spatial distribution patterns of prey species in areas with specific natural apex predators, we applied the same network robustness analysis to the individual networks of the three apex predators (dhole: n_IEs_ = 2660; Asian golden cat: n_IEs_ = 1208; clouded leopard: n_IEs_ = 962). All analyses were conducted using R 4.5.0 (R Core Team, 2025). All statistical tests were evaluated at a significance level of *p* = 0.05. For models with multiple comparisons, *p*‐values were adjusted using the Bonferroni method.

## 3 RESULTS

The 400 camera traps deployed across eastern Xishuangbanna from 2017 to 2021 yielded 113,663 trap days and 30,778 independent events (IEs). Propensity score matching identified 236 environmentally comparable sites, providing 66,679 trap days of data. Hunters were detected at 21 sites (8.9% of the total sites, average RAI = 0.58, range = 0.27–2.43), and apex predators were detected at 65 sites (27.5%, average RAI = 0.63, range = 0.27–2.35). Among single‐species detections of apex predators, dholes were most frequent (27 sites, average RAI = 0.68, range = 0.28–2.35), followed by Asian golden cats (18 sites, average RAI = 0.54, range = 0.27–1.36) and clouded leopards (10 sites, average RAI = 0.41, range = 0.27–0.62).

At the 236 matched sites, cameras recorded 17,434 independent events representing 38 animal species, including medium‐ to large‐sized mammals and ground‐dwelling birds (Table S5). Four species were prevalent: northern red muntjac (*Muntiacus vaginalis*), wild boar (*Sus scrofa*), silver pheasant (*Lophura nycthemera*), and masked palm civet (*Paguma larvata*). Twenty species were classified as common, including seven mesopredators (44% of all mesopredators), as well as ungulates, primates, ground‐dwelling birds, and rodents. Fourteen species were rare, eight of which were mesopredators, mostly from the Viverridae family (Table S5).

### 3.1 Impacts of hunters versus the entire apex predator guild on prey communities

**FIGURE 2:**
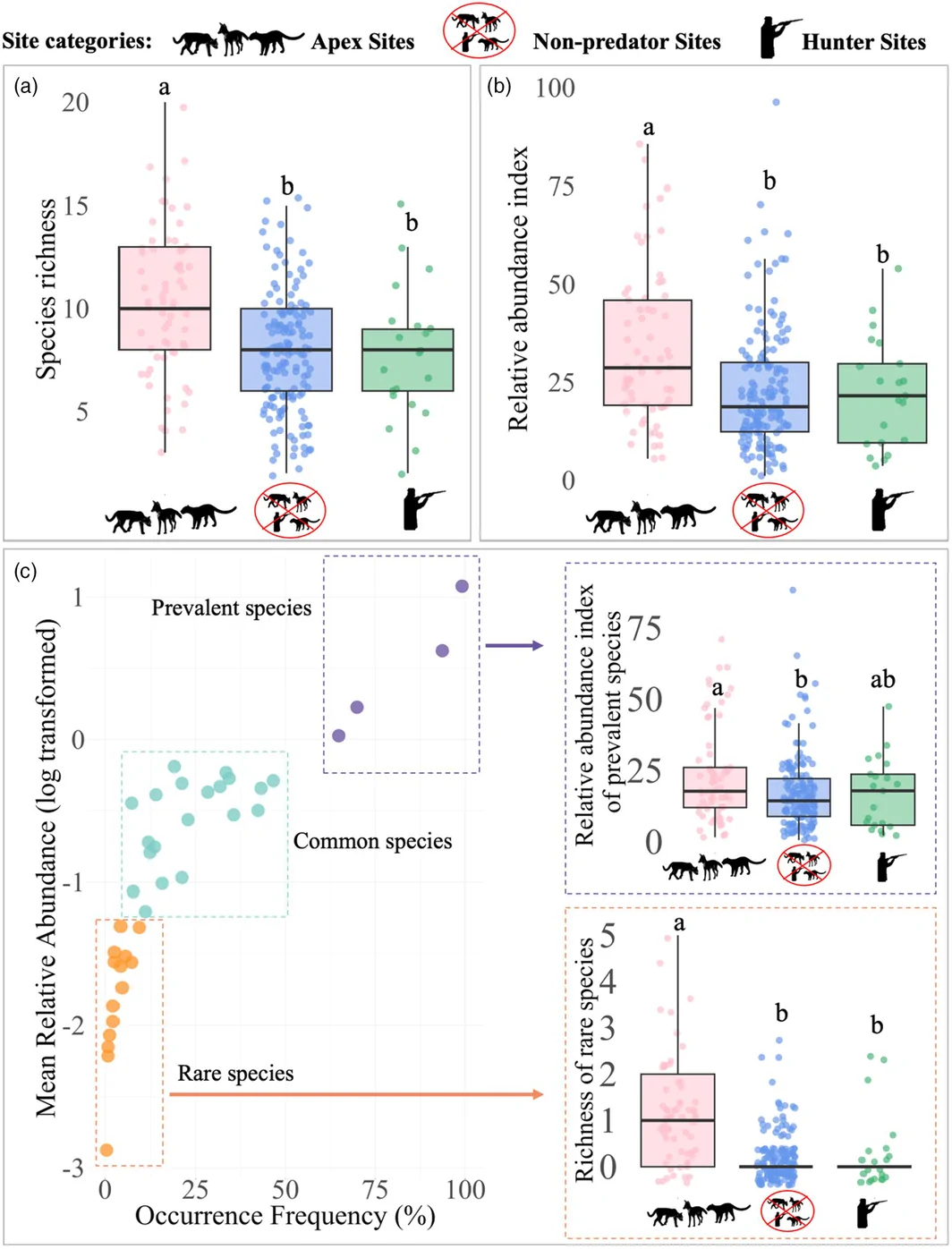
Comparison of animal community structure across three site categories (apex, hunter, and non‐predator sites) in eastern Xishuangbanna, Yunnan Province, China. (a) Total species richness and (b) total relative abundance across the three site categories, showing the highest values at apex sites. (c) Distribution of animal species in the abundance–occupancy space. Purple, green, and orange circles represent the mean relative abundance and occurrence frequency of prevalent, common, and rare species, respectively. Inset boxplots show the relative abundance of common species (top right), with apex sites having the highest values, and the richness of rare species (bottom right), also highest at apex sites. The different lowercase letters indicate significant differences between groups (*p* < 0.05; pairwise comparisons conducted following significant Kruskal–Wallis rank‐sum tests, with Bonferroni adjustment for multiple testing).

Apex predator sites showed the highest species richness, averaging 10 species per site (Kruskal–Wallis *χ* ^2^ = 24.39, df = 2, *p* < 0.001). Mean richness at these sites exceeded that of non‐predator and hunter sites by 32.5% and 33.0%, respectively (Figure 2a). Apex sites also had the highest overall abundance, with a mean RAI of 33.38 (Kruskal–Wallis *χ* ^2^ = 15.79, df = 2, *p* < 0.001), which was 44.8% higher than at non‐predator sites and 49.8% higher than at hunter sites (Figure 2b).

Reduced richness at hunter and non‐predator sites was mainly due to the loss of rare species (Kruskal–Wallis *χ* ^2^ = 62.05, df = 2, *p* < 0.001; Figure 2c). Lower abundance at non‐predator sites was associated with lower RAI values for prevalent species (Kruskal–Wallis *χ* ^2^ = 6.03, df = 2, *p* = 0.049). In contrast, the reduced abundance at hunter sites did not show a species‐specific pattern (Figure 2c).

**FIGURE 3:**
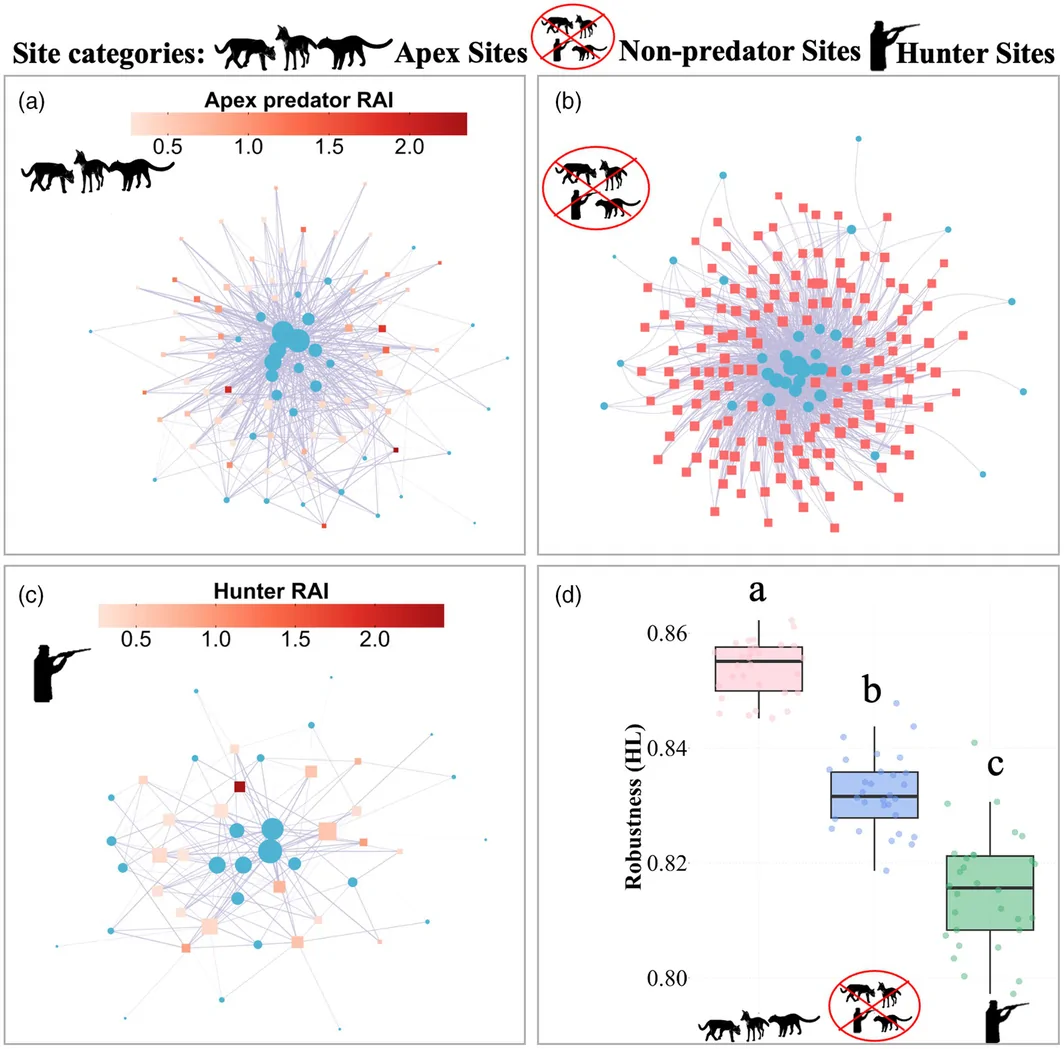
Network structures at apex, hunter, and non‐predator sites in eastern Xishuangbanna, Yunnan Province, China. (a–c) Bipartite prey species–site networks for each site category. Species (blue circles) and camera‐trap sites (squares) are sized relative to their degree centrality (the number of unique sites per species or species per site, respectively). Site nodes are further colour‐coded based on the relative abundance index (RAI) of apex predators and hunters; the gradient from pale pink to deep red represents increasing levels of predator or hunter activity. (d) Network robustness across the three categories. Different lowercase letters denote significant differences (*p* < 0.05).

Networks at apex and non‐predator sites displayed relatively even species distributions, whereas hunter sites exhibited pronounced unevenness, with species richness heavily aggregated in plots characterized by low hunter RAI (Figure 3a–c). Network robustness differed significantly among the three categories (*F*_2,87_ = 177.8, *p* < 0.001), with apex sites exhibiting the highest robustness, followed by non‐predator sites and hunter sites (Figure 3d).

### 3.2 Functional divergence: Hunter versus individual apex predator species

**FIGURE 4:**
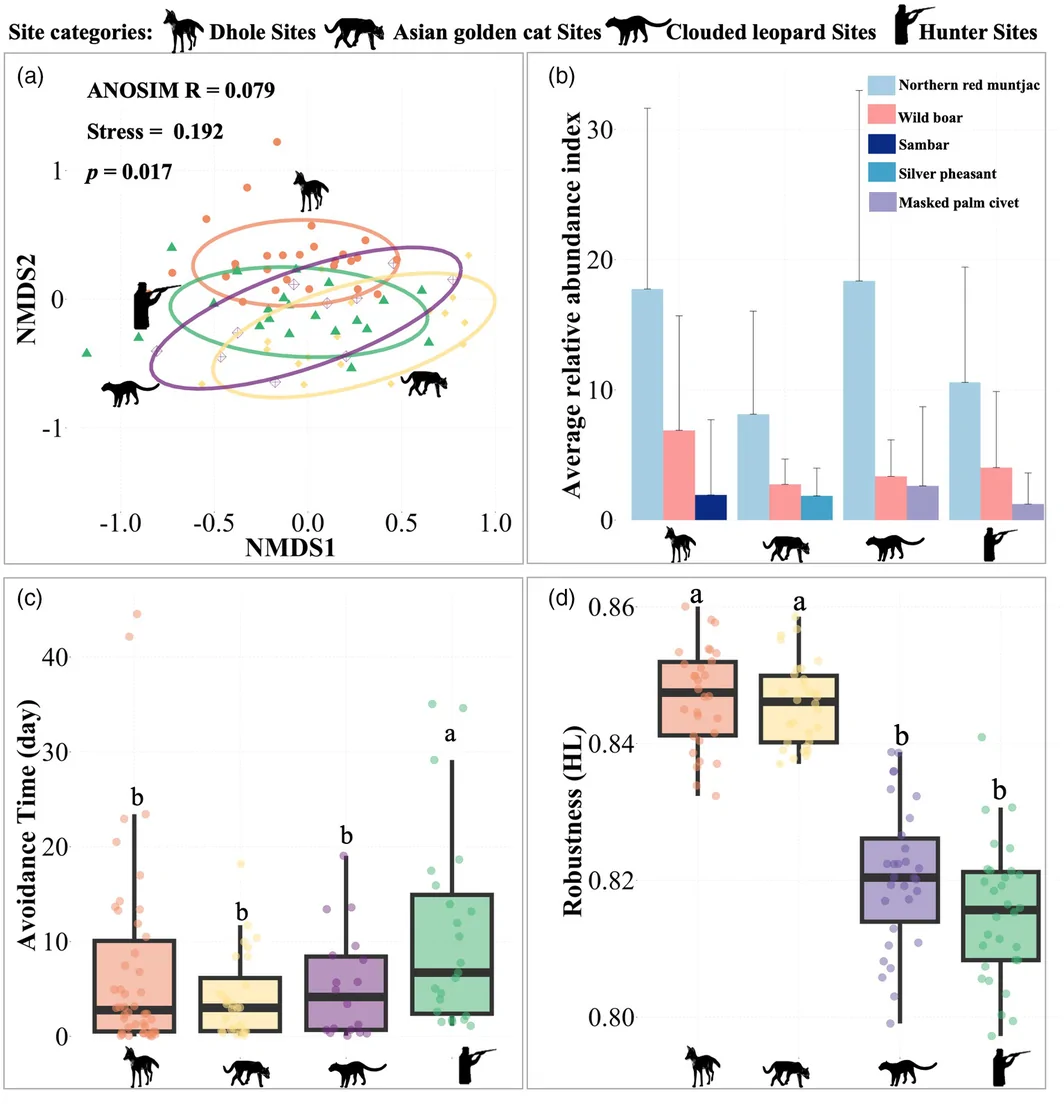
Comparison of species composition, anti‐predator behaviour, and network structures at sites of three apex predators (dhole *Cuon alpinus*, Asian golden cat *Catopuma temminckii*, and clouded leopard *Neofelis nebulosa*) and hunter sites in eastern Xishuangbanna, Yunnan Province, China. (a) Non‐metric multidimensional scaling (NMDS) ordination of species composition across hunter and three natural apex predator sites. (b) Relative abundances of the three dominant species at each site type (error bars show mean ± SD). (c) Prey avoidance times following detections of predators or hunters, defined as the interval between the detection of a predator or hunter and the subsequent prey detection at the same site. (d) Robustness of species–site networks. Different lowercase letters denote significant differences (*p* < 0.05).

Species composition varied significantly among dhole, Asian golden cat, clouded leopard, and hunter sites (ANOSIM R = 0.079, *p* = 0.017; Figure 4a). Differences were largely explained by the three dominant prey species at each site, which together accounted for an average of 53.1% of compositional variation across the four site categories (Table S6). Northern red muntjac and wild boar were consistently the top two species, whereas the third species varied: sambar at dhole sites, silver pheasant at Asian golden cat sites, and masked palm civet at both clouded leopard and hunter sites (Figure 4b).

Prey exhibited significantly shorter avoidance durations towards apex predators than towards hunters (Figure 4c). Average avoidance time was longest after hunter detections (10.7 days, SD = 10.4, range: 1.1–35.1), 1.5 times longer than for dholes (7.0 days; SD = 10.1, range: 0.003–44.5), twice as long as for clouded leopards (5.4 days; SD = 5.7, range: 0.08–19), and 2.5 times longer than for Asian golden cats (4.2 days; SD = 4.5, range: 0.09–18.2).

Network robustness also differed significantly among the four predator site types (*F*_2,87_ = 108.8, *p* < 0.001). Dhole and Asian golden cat networks showed significantly greater robustness than those of clouded leopards and hunters (Figure 4d).

## 4 DISCUSSION

Our findings demonstrate that human hunters cannot functionally replace the collective role of the natural apex predator guild, nor can they substitute for the ecological functions of individual predator species. Specifically, when comparing the roles of human hunters versus apex predator guild, we found that sites with apex predators sustained the highest species richness and abundance, whereas hunter sites failed to maintain comparable diversity, particularly among rare species. Furthermore, the presence of apex predators contributed to a more even prey distribution pattern. In contrast, the presence of hunters led to a clustered species distribution, concentrated in sites with lower hunter RAI. Regarding the distinct impacts associated with individual natural predators and hunters, species composition differed significantly among the four site types, namely sites occupied by dhole, Asian golden cat, clouded leopard, or hunters. This divergence was primarily driven by variations in the composition and abundance of dominant prey species at each site. Moreover, among all predator types, hunters elicited the strongest prey avoidance responses and the lowest robustness of prey–site networks.

When comparing human hunters with the apex predator's guild as a whole, our results demonstrate that hunters fail to achieve functional equivalence with their natural counterparts. Specifically, we observed that hunter sites exhibited significantly lower species richness, particularly among rare species, and lower overall abundance than apex sites (Figure 2). This decline likely reflects the dual impacts of hunting: high lethal mortality (Darimont et al., 2015) and a strong ‘ecology of fear’ (Clinchy et al., 2016). Under such pressures, common, fast‐reproducing species tend to persist, whereas rare and sensitive species are often extirpated or exhibit active avoidance of hunting sites. Furthermore, in protected areas where enforcement and detection risks are higher (Li, 2007), hunters are likely to adopt opportunistic behaviour, exploiting any prey they encounter immediately (Dalerum & Swanepoel, 2017). This non‐selective hunting pressure may explain why overall prey abundance declined at hunted sites without showing a consistent species‐specific pattern (Figure 2c).

Hunters' impacts are further reflected in the prey–site network structure, which exhibits a clustered and heterogeneous spatial pattern: sites with lower hunter RAI support higher species richness, whereas sites with higher hunter RAI are notably impoverished (Figure 3c). Importantly, hunter sites exhibited the lowest network robustness among all site categories (Figure 3d). This likely stems from strong hunter‐induced fear (e.g., Clinchy et al., 2016; Liu et al., 2023), which may constrain animal movement and impede dispersal capacity. For example, perceived risk drives ungulates to avoid hunter‐frequented areas, compressing their available habitat into limited low‐risk refugia (Cromsigt et al., 2013). Ultimately, such spatial concentration could undermine network robustness; any further disturbance to these critical refugia could trigger local extinctions or cascading network collapse, thereby compromising community‐wide resistance.

At non‐predator sites, species richness declined similarly to that at hunter sites, primarily due to the loss of rare species; meanwhile, the observed decrease in total abundance was driven by a reduction in prevalent species (Figure 2). This pattern suggests a mesopredator release effect resulting from the absence of apex predators (Prugh et al., 2009). Our study revealed that seven mesopredator species (44% of total mesopredators) were common, including adept predators such as the mainland leopard cat (*Prionailurus bengalensis*) and yellow‐throated marten (*Martes flavigula*) (Table S5). Without top‐down control, these mesopredators likely proliferated, suppressing other species (Ripple et al., 2013). On one hand, the high resource demands of these released mesopredators can impose intense predation pressure on common prey species (e.g., ungulates and pheasants; Kamler et al., 2020), potentially leading to a direct reduction in their abundances. On the other hand, intensified intraguild competition disproportionately affects subordinate mesopredators (Davies et al., 2021), potentially driving the observed decrease in rare species (Figure 2c)—a group that included eight mesopredator species. This competition may also prevent any one mesopredator from achieving dominance, explaining why even the prevalent mesopredator, the masked palm civet, showed no abundance advantage at non‐predator sites. Furthermore, bipartite networks at non‐predator sites displayed more uniform node sizes (Figure 3b), suggesting that prey distribution may exhibit a more spatially homogeneous structure. Unlike the broad cascading effects of intensive hunting, mesopredator release appears to influence only specific community segments, potentially resulting in intermediate network robustness (Figure 3d).

Apex predator sites sustained significantly higher species richness and abundance than both hunter and non‐predator sites (Figure 2a–c). This pattern could result from reduced direct mortality (Darimont et al., 2015) and fear (e.g., Clinchy et al., 2016; Liu et al., 2023), while also limiting mesopredator proliferation (Ritchie & Johnson, 2009). As a result, vulnerable prey could persist and coexist with species at lower trophic levels. Apex predators can also serve as umbrella species, supporting smaller animals with limited area requirements (Gittleman et al., 2001), and provide carrion for scavengers (Wilmers et al., 2003). Their presence is therefore critical for conserving rare species and sustaining broader biodiversity. Although apex predators also create landscapes of fear, their dispersed and sustainable predation strategies contrast with the intensive pressure imposed by hunters (Wilmers et al., 2003). These strategies may foster prey movement and dispersal, likely reducing dependence on specific refuge sites and buffering against local extirpation. Collectively, these processes likely sustain dynamic species–site interactions and enhance ecological connectivity, culminating in the highest network robustness observed at apex sites (Figure 3d) and highlighting their irreplaceable role in community resistance.

Beyond the guild level, clear functional divergence is also evident at the species level. Hunters fail to replicate the ecological role of any individual natural apex predator species. We found significant differences in species composition across sites occupied by the three apex predators and hunters (Figure 4a), primarily driven by variations in dominant prey species at each site. Northern red muntjac and wild boar were dominant across all predator types (Figure 4b), reflecting their roles as key prey for apex predators (Kamler et al., 2012, 2020), frequent hunting targets (Chen et al., 2019), and broad distributions (Table S5). Nonetheless, the abundance of the third dominant prey species varied distinctly among apex predators and hunters. For example, the third most dominant species differed by site: sambar (a large ungulate) at dhole sites, silver pheasant (a ground‐dwelling bird) at Asian golden cat sites, and masked palm civet (a mesopredator) at clouded leopard and hunter sites (Figure 4b).

These patterns are consistent with known predator‐specific prey profiles reported in dietary studies (e.g., Kawanishi & Sunquist, 2008; Rasphone et al., 2022; Xiong et al., 2017) and with records indicating that hunters frequently harvest masked palm civets in this region (Chen et al., 2019). This suggests that predators may exhibit distinct prey preferences and spatially track prey availability. Variation in species composition across sites occupied by the three apex predators may further reflect niche differentiation, including differences in predation strategies. For instance, dholes employ cooperative hunting tactics, enabling them to capture large ungulates such as sambar (Kamler et al., 2012). By combining arboreal diurnal activity with terrestrial nocturnality (Chiang, 2017), clouded leopards maximize their spatiotemporal overlap with smaller carnivores, thereby enhancing encounter rates and predation opportunities (Rasphone et al., 2022). In multipredator systems, such niche segregation not only promotes the coexistence of apex predators (Rasphone et al., 2020) but also enables top‐down control of different prey guilds, thereby potentially fostering community stability.

Although hunter sites also differed in species composition from apex predator sites (Figure 4a), this divergence may not stem from adaptive mechanisms such as niche differentiation. Instead, our findings suggest that these differences could be attributed to overharvesting, as evidenced by the reduced richness of rare species and lower overall community abundance observed at hunter sites (Figure 2a–c). Consequently, hunters cannot replicate the complementary prey‐regulation system in which diverse apex predators target different prey guilds. Moreover, hunters not only impose direct pressures (e.g., persecution) on apex predators (Darimont et al., 2015) but may also compete for shared resources, such as overlapping dominant prey (Figure 4b). These synergistic stressors may further undermine the persistence of apex predator populations.

The irreplaceability of apex predators is further evidenced by prey anti‐predator responses. Our study revealed significantly longer delays in prey reappearance following hunter presence compared with predator presence (Figure 4c). These findings indicate that prey species may exhibit more pronounced fear responses towards hunters than towards natural apex predators, a pattern consistent with previous studies (e.g., Clinchy et al., 2016; Liu et al., 2023). This may be attributed to the co‐evolutionary balance between prey and apex predators, which has fine‐tuned anti‐predator behaviours over time (Blumstein, 2006). Although humans have co‐evolved with wildlife for millennia and many species have adapted to anthropogenic risks (Johann et al., 2020; Thurfjell et al., 2017), modern hunters, armed with firearms and characterized by irregular activity patterns and unrestricted movement, constitute a novel, pervasive, and unpredictable threat (Darimont et al., 2015). Such unpredictability could hinder prey capacity to rapidly adapt to human‐induced risks (Vermeij, 2012), potentially resulting in markedly prolonged delays in prey reappearance following hunter activity (Figure 4c). It is noteworthy that prey avoidance durations exhibited high interspecific variability, with standard deviations approximating or exceeding their respective means. This implies substantial behavioural heterogeneity, likely reflecting diverse risk sensitivities, poaching histories, and fine‐scale habitat features that influence perceived safety (Ciuti et al., 2012). Thus, although hunters impose the greatest pressure, their impact creates a ‘landscape of fear’ that is characteristically dynamic and species‐specific.

In addition to temporal avoidance, prey–site networks at hunter sites exhibited significantly lower robustness compared with sites dominated by the dhole and the Asian golden cat (Figure 4d). Unlike human hunting, which is often intensive and spatially localized (Wilmers et al., 2003), the broader spatial range of dholes and Asian golden cats may result in a more sustainable regulatory influence. For instance, the pack‐living behaviour of dholes requires high food intake, leading them to target larger prey and forage across extensive areas (Aryal et al., 2015), consistent with our finding that dholes occupied the most sites among all apex predators. Although solitary, the Asian golden cat also maintains a wide activity range (Grassman et al., 2005). Such extensive spatial activity likely imposes fewer movement restrictions on prey, thereby preserving more robust and natural spatial distribution patterns.

Although clouded leopard sites exhibited low robustness similar to hunter sites, the underlying mechanisms may differ fundamentally. As an arboreal and nocturnal species (Chiang, 2017), the clouded leopard relies on trees for resting and hunting (Lloyd et al., 2006), resulting in limited direct regulation of prey that are temporally or spatially segregated. Thus, the low robustness associated with clouded leopard sites may reflect niche differentiation rather than the high lethality and fear‐driven restrictions characteristic of human hunting. These dynamics highlight that even within the same family, Felidae, the Asian golden cat and clouded leopard can exert distinct effects on prey distribution, underscoring the unique and irreplaceable roles of each apex predator.

Despite the insights gained from this study, some limitations should be acknowledged. First, although we employed PSM to control for key environmental variables across site categories, the potential influence of unmeasured covariates cannot be ruled out entirely. Second, the detection of hunters (e.g., via camera traps) does not necessarily equate to actual hunting pressure or successful harvest events. Future research would benefit from integrating direct mortality data (e.g., carcass surveys or telemetry) to more accurately quantify the lethal impact of hunter presence on prey species. Third, since our sampling was restricted to protected areas, potential anthropogenic pressures originating from outside the borders remained unmeasured. Boundary sites are particularly vulnerable to these external influences, especially given that hunting is often driven by accessibility to roads and villages. Future research should employ transects spanning reserve boundaries to capture the full extent of ecological gradients and assess the extent of human disturbance within protected cores. Finally, the ongoing decline of apex predator populations is likely a protracted process that has reshaped animal community composition and structure over time and will likely continue to do so in the future. Therefore, these insights could be used not only to understand the present state of these communities, but also to predict future changes.

In conclusion, our results demonstrate the unique and irreplaceable ecological functions of each apex predator and the functional integrity of the entire predator ensemble in tropical forest ecosystems. Hunters cannot functionally replicate the role of the apex predator guild or any individual apex predator species. Consequently, conservation strategies should prioritize restoring natural apex predator functionality over human‐mediated predator control. Given the endangered status of many apex predators, we recommend implementing long‐term adaptive management that integrates predator recovery programmes with strictly enforced hunting bans in critical zones.

## AUTHOR CONTRIBUTIONS

*Conceptualization*: Ying Geng, Rui‐Chang Quan; *Data curation*: Zhong‐yuan Zhang; *Funding acquisition*: Bo Wang, Lin Wang, Christos Mammides, Rui‐Chang Quan; *Methodology*: Ying Geng, Bo Wang, Ru‐Chuan He, Christos Mammides; *Visualization*: Ying Geng, Christos Mammides, Kang Luo, Rui‐Chang Quan; *Writing—original draft*: Ying Geng, Rui‐Chang Quan; *Writing—review and editing*: Ying Geng, Bo Wang, Lin Wang, Christos Mammides, Kang Luo, Rui‐Chang Quan.

## CONFLICT OF INTEREST STATEMENT

All authors declare that they have no competing interests.

## Supporting information


**Figure S1.** Camera‐trap images from the study site showing human hunters and the three natural apex predators: Top Left: Hunter (armed); Top Right: Dhole (*Cuon alpinus*); Bottom‐Left: Asian golden cat (*Catopuma temminckii*); Bottom Right: Clouded leopard (*Neofelis nebulosa*).
**Figure S2.** Density distributions of environmental covariates across the three site categories (hunter, apex, and non‐predator sites) before and after balancing. The plots illustrate that the distributions of key environmental factors have become more balanced following propensity score matching (PSM) and our iterative site selection procedure.
**Table S1.** Information on the design of the camera‐trap survey in the study area.
**Table S2.** Multivariate cutoff level analysis profiles for dataset structure.
**Table S3.** Pairwise Wilcoxon rank‐sum test results for environmental differences among three site types: hunter, apex, and non‐predator sites. *p* > 0.05 indicates no significant difference between the two compared site types.
**Table S4.** Results of Moran's *I* test for spatial autocorrelation of apex predator and hunter detections across the original 400 sites and 236 matched sites. *p* > 0.05 indicates no significant spatial autocorrelation, confirming that the 500 m minimum distance between camera traps was sufficient to ensure sampling independence for both apex predators and human hunters.
**Table S5.** Detection of animal species, along with their relative abundance index (RAI), body size, and traits in the eastern Xishuangbanna, Yunnan Province, China.
**Table S6.** Cumulative contribution of the top three species to dissimilarity in species composition between the three apex predator sites (dhole, clouded leopard, and Asian golden cat) and hunter sites.

## Data Availability

Data available from the Zenodo Digital Repository https://doi.org/10.5281/zenodo.20519006 (Geng et al., 2026).
