# Supplementary material for: Human hunters are no substitute for vanishing apex predators
Source: J Anim Ecol. 2026 Jun 26;95(9):1807–19. doi: 10.1111/1365-2656.70306 (PMC13536251; doi:10.1111/1365-2656.70306)
Supplement: Supplementary file 1 [file JANE-95-1807-s001.docx]

**Human Hunters Are No Substitute for Vanishing Apex Predators**

The Appendix offers detailed supplementary information to the main text’s Methods section, specifically covering the identification of prevalent-rare species and criteria for environmentally similar sites. Furthermore, it features two figures and six tables that provide a more in-depth elaboration on the data analysis processes. A final, dedicated section presents the supporting references.

**Supplementary information list:**

**Section 1 Identifying prevalent-rare species**

**Section 2 Site selection**

**Figure S1** Camera trap images from the study site showing human hunters and the three natural apex predators: Top-Left: Hunter (armed); Top-Right: Dhole (*Cuon alpinus*); Bottom-Left: Asian golden cat (*Catopuma temminckii*); Bottom-Right: Clouded leopard (*Neofelis nebulosa*).

**Figure S2** Density distributions of environmental covariates across the three site categories (hunter, apex, and non-predator sites) before and after balancing. The plots illustrate that the distributions of key environmental factors have become more balanced following propensity score matching (PSM) and our iterative site selection procedure.

**Table S1** Information on the design of the camera trap survey in the study area.

**Table S2** Multivariate cutoff level analysis profiles for dataset structure.

**Table S3** Pairwise Wilcoxon rank-sum test results for environmental differences among three site types: hunter, apex, and non-predator sites. *p* > 0.05 indicates no significant difference between the two compared site types.

**Table S4** Results of Moran’s I test for spatial autocorrelation of apex predator and hunter detections across the original 400 sites and 236 matched sites. *p* > 0.05 indicates no significant spatial autocorrelation, confirming that the 500 m minimum distance between camera traps was sufficient to ensure sampling independence for both apex predators and human hunters.

**Table S5** Detection of animal species, along with their relative abundance index (RAI), body size, and traits in the eastern Xishuangbanna, Yunnan Province, China.

**Table S6** Cumulative contribution of the top three species to dissimilarity in species composition between the three apex predator sites (dhole, clouded leopard, and Asian golden cat) and hunter sites.

**Section I Identifying prevalent-rare species**

In this study, we employed multivariate cutoff level analysis (Gobet et al., 2010) to identify prevalent and rare species in the community. This method systematically removes species based on cumulative relative abundance thresholds, ranging from 1% to 99%. For each cutoff level, we computed the Spearman correlation between the original species matrix and truncated matrix using Bray–Curtis dissimilarity to evaluate how well the reduced community represented the original structure.

The analysis revealed that removing species comprising the cumulative 1% of relative abundance (corresponding to 13 species) resulted in a truncated matrix maintaining 99% similarity with the original community. The remaining 25 species accounted for 99% of the total relative abundance, initially identifying the 13 excluded species as the rarest (Table S2). At the 25% cutoff level (removing species comprising 25% of cumulative relative abundance), only four species remained, yet the truncated matrix maintained high similarity with the original (Spearman correlation = 0.91). These four species collectively accounted for over 70% of the total relative abundance (Table S2).

Based on these analytical results and incorporating occurrence frequency criteria, we established the following classification: prevalent species were defined as those observed in more than 60% of sampling sites with a mean relative abundance exceeding 1.0, which aligned with the four species identified at the 25% cutoff level (Table S2). Rare species were characterized by a mean relative abundance below 0.05 and occurrence in fewer than 10% of sites. This definition captured the 13 species identified through the 1% cutoff analysis, plus one additional species with similarly low abundance characteristics but exceptionally low occurrence frequency (4.2%), resulting in a final designation of 14 rare species (Table S5). Species falling between these thresholds were categorized as common (Table S5).

This cutoff-based approach, when integrated with species occurrence frequency, established a robust framework for identifying the ecological significance of prevalent and rare species. This integrated framework pragmatically incorporates critical dimensions, including ecological function, conservation priority, restorative difficulty, and dynamics of species interactions (e.g., Estes et al., 2011; Harrison et al., 2016).

**Section II Site selection**

To study the impact of predation pressure on species richness, abundance, composition, and distribution, we divided our 400 camera sites into three categories based on the distribution of the natural apex predators and hunters: (1) Apex Sites (n_sites_ = 81), i.e., sites where apex predators were detected but hunters were absent. (2) Hunter Sites (n_sites_ = 21), i.e., sites where hunters were detected but apex predators were absent. (3) Non-predator Sites (n_sites_ = 288), used as the control group and composed of sites where neither hunters nor apex predators were detected. To isolate the effects of predation risk from confounding environmental factors, we selected camera sites with similar environmental characteristics.

First, we used pairwise Wilcoxon tests to compare environmental factors across our three site categories: hunter, apex, and non-predator sites. The factors included tree density, normalized difference vegetation index, slope, aspect, topographic position index, distances to roads, distances to plantations, distances to water surfaces, and the relative abundance index (RAI) of villagers and livestock. Our analysis revealed no significant environmental differences between hunter and non-predator sites (*p* > 0.05). However, we found significant differences in the following comparisons: Hunter vs. Apex sites—tree density, distances to roads, and distances to plantations; and Apex vs. Non-predator sites—tree density, distances to water surfaces, distances to plantations, and the RAI of villagers.

To address these imbalances, we first employed propensity score matching using a generalized linear model (GLM) with a nearest-neighbor, no-replacement algorithm. This procedure, which used hunter sites as the control group, was based on tree density; distances to water surfaces, roads, and plantations; and the RAI of villagers. This initial step selected 66 apex sites and 249 non-predator sites. However, a subsequent check showed that significant differences in distances to water surfaces and the RAI of villagers still persisted between the new apex and non-predator sites (*p* < 0.05).

To fully resolve this remaining imbalance, we initiated a more targeted balancing process. Because the mean distances to water surfaces and RAI of villagers were lower at the non-predator sites than at the apex sites, we focused on adjusting the non-predator group. We sequentially removed non-predator sites with low distances to water and low RAI of villagers until the disparity in these factors was no longer significant between the non-predator and apex sites.

During this process, a new significant difference in tree density emerged between the hunter and non-predator sites. To correct this, we again sorted the remaining non-predator sites, this time by tree density and the RAI of villagers. We then sequentially removed sites with high tree density and low RAI of villagers until no significant environmental differences remained among any of the three site categories.

Ultimately, this iterative balancing procedure resulted in a final sample of 21 hunter sites, 65 apex sites, and 150 non-predator sites. A final check confirmed no significant differences in any environmental factor among the three site categories (*p* > 0.05), ensuring our groups were environmentally comparable (Table S3).

In total, we excluded 16 apex sites and 138 non-predator sites from the analysis. The excluded sites had an overall mean species richness of 8.7 and a mean RAI of 25.8. Specifically, the excluded apex predator sites exhibited a mean richness of 11.3 and an RAI of 38.9, while the excluded non-predator sites showed a mean richness of 8.2 and an RAI of 23.9.

**Figure S1** Camera trap images from the study site showing human hunters and the three natural apex predators: Top-Left: Hunter (armed); Top-Right: Dhole (*Cuon alpinus*); Bottom-Left: Asian golden cat (*Catopuma temminckii*); Bottom-Right: Clouded leopard (*Neofelis nebulosa*).

**Figure S2** Density distributions of environmental covariates across the three site categories (hunter, apex, and non-predator sites) before and after balancing. The plots illustrate that the distributions of key environmental factors have become more balanced following propensity score matching (PSM) and our iterative site selection procedure.

**Table S1** Information on the design of the camera trap survey in the study area.

| Set up year | No. camera-trapping sites | No. transects | Surveyed area |
| --- | --- | --- | --- |
| 2017 | 38 | 35 | Menglun / Mengla / Yiwu |
| 2018 | 57 | 41 | Menglun / Mengla / Yiwu / Shangyong |
| 2019 | 165 | 63 | Menglun / Mengla / Yiwu / Shangyong / Mengyang |
| 2020 | 140 | 44 | Menglun / Yiwu / Shangyong / Mengyang |

**Table S2** Multivariate cutoff level analysis profiles for dataset structure.

| Cutoff level (%) | Spearman correlation | Remaining species | Abundance retained percent |
| --- | --- | --- | --- |
| 0 | 1 | 38 | 100 |
| 1 | 0.998973839 | 25 | 98.99714378 |
| 5 | 0.9913728 | 16 | 94.32489823 |
| 10 | 0.977848813 | 12 | 88.97357915 |
| 15 | 0.965105716 | 9 | 83.74549115 |
| 20 | 0.944679323 | 7 | 79.84839516 |
| 25 | 0.912188126 | 4 | 73.00427822 |
| 30 | 0.892852573 | 3 | 68.88918936 |
| 35 | 0.848551401 | 2 | 62.36341751 |
| 40 | NA | 1 | 46.10526153 |
| 45 | NA | 1 | 46.10526153 |
| 50 | NA | 1 | 46.10526153 |
| 55 | NA | 1 | 46.10526153 |
| 60 | NA | 1 | 46.10526153 |
| 65 | NA | 1 | 46.10526153 |
| 70 | NA | 1 | 46.10526153 |
| 75 | NA | 1 | 46.10526153 |
| 80 | NA | 1 | 46.10526153 |
| 85 | NA | 1 | 46.10526153 |
| 90 | NA | 1 | 46.10526153 |
| 95 | NA | 1 | 46.10526153 |
| 99 | NA | 1 | 46.10526153 |

**Table S3** Pairwise Wilcoxon rank-sum test results for environmental differences among three site types: hunter, apex, and non-predator sites. *p* > 0.05 indicates no significant difference between the two compared site types.

| Variables | Apex sites versus non-predator sites (*p*) | Hunter sites versus non-predator sites (*p*) | Apex sites versus hunter sites (*p*) |
| --- | --- | --- | --- |
| Tree density | 1.000 | 0.158 | 0.073 |
| Normalized difference vegetation index | 1.000 | 0.62 | 0.86 |
| Slope | 1.000 | 0.27 | 0.51 |
| Aspect | 1.000 | 0.22 | 0.80 |
| Topographic position index | 0.16 | 0.75 | 1.000 |
| Distance to water | 0.67 | 0.35 | 0.83 |
| Distance to road | 1.00 | 0.46 | 0.44 |
| Distance to plantation | 1.00 | 0.42 | 0.31 |
| Relative abundance of livestock | 0.77 | 0.85 | 0.46 |
| Relative abundance of villager | 0.067 | 1.000 | 1.000 |

**Table S4** Results of Moran’s *I* test for spatial autocorrelation of apex predator and hunter detections across the original 400 sites and 236 matched sites. *p* > 0.05 indicates no significant spatial autocorrelation, confirming that the 500 m minimum distance between camera traps was sufficient to ensure sampling independence for both apex predators and human hunters.

|  | 400 sites | | 236 matched sites | |
| --- | --- | --- | --- | --- |
|  | Moran’s *I* statistic | *p* | Moran’s *I* statistic | *p* |
| Apex predators | 0.153 | 0.060 | 0.088 | 0.212 |
| Hunters | -0.006 | 0.488 | 0.021 | 0.382 |

**Table S5** Detection of animal species, along with their relative abundance index (RAI), body size, and traits in the eastern Xishuangbanna, Yunnan Province, China.

| Order | Family | Scientific name | Common name | IUCN red list^1^ | Mean RAI across 236 camera sites | Detected camera sites | Body weight (kg)^2^ | Species categories | Traits^3^ |
| --- | --- | --- | --- | --- | --- | --- | --- | --- | --- |
| Cetartiodactyla | Cervidae | *Muntiacus vaginalis* | Northern Red Muntjac | LC | 11.909 | 234 | 17-40 | Prey /  Prevalent | Large / Terrestrial / Herbivore / Habitat generalist / Seed disperser / Major prey of big carnivores |
| Cetartiodactyla | Suidae | *Sus scrofa* | Wild Boar | LC | 4.199 | 221 | 50-200 | Prey /  Prevalent | Large / Terrestrial / Omnivorous / Habitat generalist / Major prey of big carnivores |
| Galliformes | Phasianidae | *Lophura nycthemera* | Silver Pheasant | LC | 1.686 | 165 | 5-12 | Prey /  Prevalent | Medium / Omnivorous / Habitat generalist |
| Carnivora | Viverridae | *Paguma larvata* | Masked Palm Civet | LC | 1.063 | 153 | 3-7 | Mesopredator /  Prevalent | Medium / Frugivorous / Mesopredator / Habitat generalist |
| Cetartiodactyla | Cervidae | *Rusa unicolor* | Sambar | VU | 0.647 | 45 | 185-260 | Prey / Common | Large / Terrestrial / Herbivore / Habitat generalist / Major prey of big carnivores |
| Rodentia | Hystricidae | *Hystrix brachyura* | Malayan Porcupine | LC | 0.587 | 79 | 1-1.5 | Prey / Common | Small / Rodent / Habitat generalist |
| Galliformes | Phasianidae | *Gallus gallus* | Red Junglefowl | LC | 0.534 | 81 | 0.8-1.1 | Prey / Common | Small / Omnivorous / Habitat generalist |
| Carnivora | Felidae | *Prionailurus bengalensis* | Mainland Leopard Cat | LC | 0.514 | 110 | 1.5-5 | Mesopredator / Common | Medium / Carnivore / Mesopredator / Habitat generalist |
| Galliformes | Phasianidae | *Polyplectron bicalcaratum* | Grey Peacock-Pheasant | LC | 0.492 | 50 | 0.6-0.7 | Prey / Common | Small / Omnivorous |
| Carnivora | Viverridae | *Paradoxurus hermaphroditus* | Common Palm Civet | LC | 0.468 | 75 | 2.4-4 | Mesopredator / Common | Medium / Carnivore / Partly arboreal / Omnivorous / Habitat generalist |
| Primates | Cercopithecidae | *Macaca leonina* | Northern Pig-Tailed Macaque | VU | 0.455 | 102 | 11-14 | Prey / Common | Medium / Arboreal / Frugivorous / Primate / Habitat generalist |
| Carnivora | Mustelidae | *Melogale moschata* | Small-Toothed Ferret Badger | LC | 0.428 | 67 | 0.5-1.6 | Mesopredator / Common | Small / Carnivore / Habitat generalist |
| Rodentia | Hystricidae | *Atherurus macrourus* | Asiatic Brush-Tailed Porcupine | LC | 0.410 | 33 | 2-4 | Prey / Common | Medium / Rodent / Habitat specialist / Fossorial |
| Cetartiodactyla | Tragulidae | *Tragulus williamsoni* | Williamson’s Chevrotain | DD | 0.358 | 17 | 2.5-4.5 | Prey / Common | Medium / Terrestrial / Herbivore |
| Carnivora | Mustelidae | *Martes flavigula* | Yellow-Throated Marten | LC | 0.318 | 100 | 0.8-2.8 | Mesopredator / Common | Small / Carnivore / Mesopredator / Arboreal / Habitat generalist |
| Carnivora | Herpestidae | *Herpestes urva* | Crab-Eating Mongoose | LC | 0.296 | 84 | 1-2.3 | Mesopredator / Common | Small / Carnivore / Omnivorous / Habitat generalist |
| Cetartiodactyla | Bovidae | *Capricornis milneedwardsii* | Mainland Serow | VU | 0.274 | 54 | 85-140 | Prey / Common | Large / Terrestrial / Herbivore / Habitat generalist |
| Galliformes | Phasianidae | *Arborophila brunneopectus* | Bar-Backed Partridge | LC | 0.191 | 28 | 0.2-0.3 | Prey / Common | Small / Omnivorous |
| Carnivora | Mustelidae | *Arctonyx collaris* | Greater Hog Badger | VU | 0.176 | 32 | 9.7-12.5 | Mesopredator | Medium / Terrestrial / Carnivore / Vermivorous / Habitat generalist |
| Primates | Cercopithecidae | *Macaca mulatta* | Rhesus Monkey | LC | 0.161 | 29 | 5-10 | Prey / Common | Medium / Arboreal and terrestrial / Omnivorous / Primate / Habitat generalist |
| Carnivora | Ursidae | *Ursus thibetanus* | Asiatic Black Bear | VU | 0.108 | 50 | 54-240 | Mesopredator / Common | Large / Carnivore / Mesopredator / Omnivorous / Seed disperser / Habitat generalist |
| Carnivora | Canidae | *Cuon alpinus* | Dhole | EN | 0.098 | 37 | 10-20 | Apex predator / Common | Medium / Carnivore / Top predator / Habitat generalist |
| Proboscidea | Elephantidae | *Elephas maximus* | Asian Elephant | EN | 0.086 | 18 | 2720-4160 | Prey / Common | Large / Terrestrial / Herbivore / Habitat generalist |
| Primates | Cercopithecidae | *Macaca assamensis* | Assamese Macaque | NT | 0.063 | 26 | 5-12 | Prey / Common | Medium / Arboreal and terrestrial / Folivorous / Primate / Habitat generalist |
| Carnivora | Viverridae | *Viverricula indica* | Small Indian Civet | LC | 0.050 | 10 | 1.6-4 | Mesopredator / Rare | Medium / Carnivore / Mesopredator / Habitat generalist |
| Carnivora | Felidae | *Catopuma temminckii* | Asian Golden Cat | VU | 0.049 | 22 | 9-16 | Apex predator / Rare | Medium / Carnivore / Top predator / Habitat generalist |
| Galliformes | Phasianidae | *Tropicoperdix chloropus* | Green-Legged Partridge | LC | 0.033 | 6 | 0.25-0.3 | Prey / Rare | Small / Omnivorous / Habitat generalist |
| Carnivora | Prionodontidae | *Prionodon pardicolor* | Spotted Linsang | LC | 0.031 | 13 | 4.1-8 | Mesopredator / Rare | Medium / Carnivore / Mesopredator / Partly arboreal / Opportunistic scavenger / Habitat generalist |
| Galliformes | Phasianidae | *Arborophila rufogularis* | Rufous-Throated Partridge | LC | 0.028 | 6 | 0.2-0.3 | Prey / Rare | Small / Omnivorous / Habitat generalist |
| Carnivora | Felidae | *Neofelis nebulosa* | Clouded Leopard | VU | 0.028 | 17 | 16-32 | Apex predator / Rare | Medium / Carnivore / Top predator / Habitat generalist |
| Rodentia | Spalacidae | *Rhizomys sumatrensis* | Indomalayan Bamboo Rat | LC | 0.026 | 10 | 2.2-4 | Prey / Rare | Medium / Rodent / Habitat generalist |
| Carnivora | Viverridae | *Arctictis binturong* | Binturong | VU | 0.018 | 11 | 9-14 | Mesopredator / Rare | Medium / Carnivore / Mesopredator / Arboreal / Habitat generalist |
| Carnivora | Mustelidae | *Mustela strigidorsa* | Stripe-Backed Weasel | LC | 0.014 | 5 | 0.4-1.2 | Mesopredator / Rare | Small / Carnivore / Mesopredator / Habitat generalist |
| Carnivora | Felidae | *Pardofelis marmorata* | Marbled Cat | LC | 0.011 | 5 | 1.5-5 | Mesopredator / Rare | Medium / Carnivore / Mesopredator / Habitat generalist |
| Carnivora | Viverridae | *Chrotogale owstoni* | Owston's Civet | EN | 0.009 | 3 | 2.4-3.4 | Mesopredator / Rare | Medium / Carnivore / Mesopredator / Habitat generalist / Invertebrate |
| Carnivora | Viverridae | *Viverra zibetha* | Large Indian Civet | LC | 0.007 | 2 | 3.4-9.2 | Mesopredator / Rare | Medium / Carnivore / Mesopredator / Habitat generalist / Invertebrate |
| Pholidota | Manidae | *Manis pentadactyla* | Chinese Pangolin | CR | 0.006 | 2 | 3.6 | Prey / Rare | Medium / Terrestrial / Habitat generalist / Invertebrate |
| Carnivora | Ursidae | *Helarctos malayanus* | Sun Bear | VU | 0.001 | 1 | 25-55 | Mesopredator / Rare | Large / Carnivore / Mesopredator / Omnivorous / Habitat generalist |

^1^ LC (Least Concern), NT (Near Threatened), VU (Vulnerable), EN (Endangered).

^2^ The body weight data for mammals and birds were sourced from Liu et al. (2020) and Wang et al. (2021), respectively.

^3^ We summarize the characteristics of mammal species using information from the IUCN Red List. For mammal species not listed on the IUCN Red List, we refer to Liu et al. (2020). For bird characteristics, information is derived from Wang et al. (2021).

**Table S6** Cumulative contribution of the top three species to dissimilarity in species composition between the three apex predator sites (dhole, clouded leopard, and Asian golden cat) and hunter sites.

| Pair group | Rank | Species | Cumulative contribution |
| --- | --- | --- | --- |
| Dhole vs. Clouded Leopard | 1 | Northern Red Muntjac | 0.334 |
| Dhole vs. Clouded Leopard | 2 | Wild Boar | 0.472 |
| Dhole vs. Clouded Leopard | 3 | Silver Pheasant | 0.54 |
| Dhole vs. Asian Golden Cat | 1 | Northern Red Muntjac | 0.306 |
| Dhole vs. Asian Golden Cat | 2 | Wild Boar | 0.435 |
| Dhole vs. Asian Golden Cat | 3 | Sambar | 0.485 |
| Dhole vs. Hunter | 1 | Northern Red Muntjac | 0.351 |
| Dhole vs. Hunter | 2 | Wild Boar | 0.51 |
| Dhole vs. Hunter | 3 | Sambar | 0.56 |
| Clouded Leopard vs. Asian Golden Cat | 1 | Northern Red Muntjac | 0.35 |
| Clouded Leopard vs. Asian Golden Cat | 2 | Wild Boar | 0.436 |
| Clouded Leopard vs. Asian Golden Cat | 3 | Masked Palm Civet | 0.515 |
| Clouded Leopard vs. Hunter | 1 | Northern Red Muntjac | 0.395 |
| Clouded Leopard vs. Hunter | 2 | Wild Boar | 0.515 |
| Clouded Leopard vs. Hunter | 3 | Masked Palm Civet | 0.597 |
| Asian Golden Cat vs. Hunter | 1 | Northern Red Muntjac | 0.313 |
| Asian Golden Cat vs. Hunter | 2 | Wild Boar | 0.424 |
| Asian Golden Cat vs. Hunter | 3 | Silver Pheasant | 0.489 |

**References**

Estes, J. A., Terborgh, J., Brashares, J. S., Power, M. E., Berger, J., Bond, W. J., et al. (2011). Trophic downgrading of planet Earth. *Science*, 333, 301–306. <https://doi.org/10.1126/science.1205106>

Gobet, A., Quince, C., & Ramette, A. (2010). Multivariate cutoff level analysis (MultiCoLA) of large community data sets. *Nucleic Acids Research*, 38, e155–e155. <https://doi.org/10.1093/nar/gkq545>

Harrison, R. D., Sreekar, R., Brodie, J. F., Brook, S., Luskin, M., O’Kelly, H., et al. (2016). Impacts of hunting on tropical forests in Southeast Asia. *Conservation Biology*, 30, 972–981. <https://doi.org/10.1111/cobi.12785>

International Union for Conservation of Nature. (2022). The IUCN Red List of Threatened Species. Available at: https://www.iucnredlist.org.

Liu. S. -Y., Wu, Y. & Li, S. (2020). Handbook of Mammals of China, second version. The Strait Publishing and Distributing Group, Fuzhou. (in Chinese)

Wang, Y. -P., Song, Y. -F., Zhong, Y. -X., Chen, C. -W. Zhao, Y. -H., Zeng, D., et al. (2021). A dataset on the life-history and ecological traits of Chinese birds. *Biodiversity Science*, 29, 1149–1153 (in Chinese). <https://doi.org/10.17520/biods.2021201>
